# Mass spectrometry imaging reveals flavor distribution in edible mushrooms

**DOI:** 10.1007/s13197-023-05883-0

**Published:** 2023-11-13

**Authors:** Mudita Vats, Berta Cillero-Pastor, Bryn Flinders, Eva Cuypers, Ron M. A. Heeren

**Affiliations:** 1 Maastricht MultiModal Molecular Imaging Institute (M4i), Division of Imaging Mass Spectrometry, Maastricht University, 6229 ER Maastricht, The Netherlands; 2 Department of Cell Biology-Inspired Tissue Engineering (cBITE), MERLN Institute for Technology-Inspired Regenerative Medicine, Maastricht University, Maastricht, The Netherlands

**Keywords:** Mushroom, MALDI-MSI, On-tissue derivatization, Flavor compounds, Food, Taste

## Abstract

The spatial distribution of molecules and compounds responsible for the flavor profile of edible button mushrooms (*Agaricus bisporous*) has never been determined. The food industry is interested in knowing the localization of these compounds. Such knowledge would enable extraction of flavor compounds from a particular regions of the mushroom, which is safer for consumption compared to alternatives such as synthetic flavoring agents. The present study utilizes matrix-assisted laser desorption/ionization-mass spectrometry imaging (MALDI-MSI), to determine the spatial distribution of flavor compounds in a mushroom. As MALDI-MSI requires very thin sections, a sample preparation protocol was optimized and sectioning fresh frozen mushrooms at 35 µm thickness was considered the best method to evaluate the distribution of flavor compounds. Further, the effect of heat on the spatial distribution of flavor compounds was investigated by heating whole mushrooms to 140 ℃ prior to sectioning. Heating reduced the water content of the mushroom and thus enabled the generation of even-thinner 17 µm thick sections. MALDI-MSI measurements performed on underivatized and on-tissue derivatized fresh frozen and heat-treated mushroom sections elucidated the spatial distribution of several flavor-related compounds.

**Supplementary Information:**

The online version contains supplementary material available at 10.1007/s13197-023-05883-0.

## Introduction

*Agaricus bisporous*, commonly known as the button mushroom, belongs to the kingdom fungi and phylum Basidiomycota. It has a pileus or cap, which is shaped like an umbrella, a stipe commonly called a stem and hymenium or gills. The flavor profile of mushrooms is classified into sweet, sour, salty, bitter and umami. The molecular compounds responsible for these flavors are free amino acids, soluble sugars, 5’ nucleotides, organic acids and salts (Sasaki et al. 1988; Lizárraga-Guerra and López 1996; Kasuga et al. 1999; Muresan et al. 2000). In addition to these molecules, lipids also contribute to the taste of mushrooms. Although heating can enhance the flavor and aroma of lipids, their oxidation can lead to the development of off-flavors.

Multiple studies have analyzed the molecular-flavor relationship of compounds in mushrooms using techniques such as LC–MS (Liquid chromatography-mass spectrometry), NMR (Nuclear magnetic resonance) (Rotzoll et al. 2005; Ming et al. 2014; Chen et al. 2015; Mittermeier et al. 2018), and GC/MS (Gas chromatography-mass spectrometry) (Chen et al. 2018). A common feature of these studies is that they relied on homogenized mushrooms. As such, information about the specific distribution of compounds was not obtained in these studies due to the homogenization of samples. Localization of taste and aroma modulating compounds, and their preservation in certain anatomical structures or release of these compounds at different stage of development, environmental or preparation conditions, is of interest to both scientists and food industry. The spatial distribution provides insight in the spatial biology of fungal development and can be utilized for the optimization of the extraction of natural flavor enhancement compounds from certain areas of the fungal fruit bodies.

The spatial distribution of a wide range of compounds present on a sample surface can be studied with mass spectrometry imaging (MSI). Matrix-assisted laser desorption/ionization-mass spectrometry imaging is the most common MSI technique (Yoshimura and Zaima 2020) (Maciel et al. 2023) (Boughton et al. 2016), which uses a UV laser that hits the surface of the sample and analyzes the molecular ions liberated at each spot or pixel. To facilitate MALDI-MSI analysis, the sample surface is coated with a UV-absorbing matrix, which also extracts molecules from the sample surface. This process retrieves the mass spectrum from each pixel and reconstructs an image, displaying the spatial distribution of metabolites, lipids, proteins, and other molecules. MSI has proved to be helpful in mapping the distribution of molecules in food samples, for instance, LAESI (laser ablation electrospray ionization) has been used to analyze the pesticides on maize, ergot, lemon, apples, oranges and cherry tomato (Nielen and van Beek 2014; Ming et al. 2014).MALDI-MSI (matrix-assisted laser desorption/ionization) has been used to assess the authenticity of beef origin, profiling of cocoa content, visualization of metabolic distribution in barley roots and analysis of the 1 mm thick sections of mushroom soft rot pathogen using a tape transfer method (Zaima et al. 2011; de Oliveira et al. 2018; Graupner et al. 2012; Sarabia et al. 2018). Moreover, the localization of mercury in mushrooms cultivated in Hg-pollution sites has been studied using LA-ICP-MS (laser ablation inductively coupled mass spectrometry) (Kavčič et al. 2019). In this study, the authors investigated the bioavailability by feeding the mushrooms that contain Hg and Se to Spanish slugs. The slugs were sacrificed and dissected for ICP-MS experiments, revealing Hg and Se-induced toxicity.LA-ICP MSI has also been applied to investigate Coptis chinensis Franch, an important medicinal plant in China, for the spatial mapping of elements such as Cr, Fe, Mn, Ca, and Zn (Huang et al. 2018). The images indicated that Cr, Mn, and Ca are preferentially accumulated in the cortex and specific vascular bundles. Moreover, there was an even distribution of P and Cu throughout the entire petiole cross-sections. In addition, calcium (Ca), magnesium (Mg), sodium (Na), potassium (K), silicon (Si), and aluminum (Al) were visualized in different structures of Populus tremuloides plant roots using ToF–SIMS-MSI (Martin et al. 2004). However, to the authors’ knowledge, mass spectrometry imaging has not been utilized for the study of the molecular distributions of flavor-related (and other) compounds in edible mushrooms. A mushroom is heterogeneous and contains a high amount of water (approximately 92%), which makes frozen sample preparation challenging. The high water content will result in the formation of ice crystals upon freezing and this causes freeze fracturing during sectioning. Herein, we present a sample preparation strategy for the analysis of molecular distributions in mushroom sections. Moreover, we use the developed strategy to map the distribution of flavor compounds in different anatomical areas of mushroom samples.

## Material and methods

### Chemicals and materials

Methanol (ULC/MS-CC/SFC grade) and chloroform (HPLC grade) were purchased from Biosolve BV (Valkenswaard, The Netherlands). All MALDI matrices, 2, 5-dihydroxybenzoic acid ≥ 98% (DHB), norharmane, N-(1-naphthyl) ethylenediamine dihydrochloride (NEDC), carboxymethyl cellulose (CMC) and amino acid standards were purchased from Sigma Aldrich. Conductive indium tin oxide (ITO)–coated glass slides were purchased from Delta Technologies (Loveland, CO, USA), TAHS (p-N,N,N- trimethylamonioanilyl N-hydroxysuccinimidylcarbamate iodide) was synthesized as described elsewhere (Arts et al. 2017).

### Sample preparation

Fresh mushrooms (white *Agaricus bisporous*) were purchased from a local supermarket. Two different analytical strategies were evaluated: mushroom stamping and mushroom sectioning.

### Mushroom stamping

A quick method of stamping/imprinting was tested for the rapid analysis of mobile compounds present in the sample. A whole mushroom was cut in half. The flat side of the cut mushroom half was pressed perpendicular against an ITO slide for a few seconds. Subsequently the half-mushroom was lifted at room temperature, leaving a molecular imprint on the ITO substrate without the complex mushroom matrix.

### Mushroom sectioning

Standard cryosectioning procedures caused freeze fracturing of the mushroom. Because of this, an objective of sectioning was to obtain unfractured thin sections to map the molecular profile of flavor compounds in the mushrooms. Cryosectioning allows the preparation of very thin sections (range of micrometers) for MALDI-MSI. It maintains molecular integrity and tissue morphology. It is also crucial to have flat samples for MALDI-MSI measurements to avoid fluctuations in signal intensities. For sectioning fresh frozen mushrooms, the half cut mushrooms were snap frozen in liquid nitrogen and mounted on a cryotome sample holder with miliQ water. Sectioning was carried out using a Leica CM3050 UV cryotome (Leica Microsystems, Wetzlar, Germany) at − 21℃ with thickness range between 10 µm to 35 µm. 35 µm thick sections were thaw mounted on ITO slides and stored at − 80℃ until further analysis.

### Embedding

An evaluation of the benefit of an embedding procedure was conducted prior to sectioning. A fresh mushroom was embedded in an aqueous mixture of 2% CMC for this purpose. Cryosectioning was performed at various thickness (15 µm to 35 µm) at − 20℃. However, the CMC block and mushroom worsened the structural integrity and no sections were obtained. As a result this method was not further investigated.

### Heat-treatment

Heat treatment is known to cause alterations in appearance, distribution of some compounds and the aroma. To visualize these alterations in the mushroom upon heating, whole mushrooms were incubated in an oven for 15 min at 140 ℃. The temperature was chosen following (Mittermeier et al. 2018). The mushrooms were environmentally cooled to room temperature and subsequently snap frozen in liquid nitrogen. For cryosectioning, a mushroom was ice-mounted on a cryotome sample holder using miliQ water. 17 µm thick sections were cut at − 20 ℃ and thaw mounted on the conductive side of an ITO slide.

### On-tissue derivatization for visualizing flavor compounds

On-tissue derivatization using TAHS helps to detect amino acids by improving the ionization efficiency and sensitivity of detection. The fresh-frozen and heat-treated mushroom sections mounted on ITO slide were dried in a vacuum desiccator for 15 min. The section was coated with a TAHS solution of 1.25 mg/mL in acetonitrile using a HTX M3 + sprayer (HTX Technologies LLC, Carrboro). Six layers were sprayed at 55 ℃ with a constant flow rate of 0.1 mL/min and at a speed of 1200 mm/min as described elsewhere (Cao et al. 2021). The mushroom section was subsequently incubated at 55 ℃ in a humid environment (methanol: water = 1:1, v/v) for 24 h. Derivatization with TAHS was also performed on some amino acid standards to confirm the identities and compare with mushroom sections. Each standard with a concentration of 0.5 mg/mL was mixed with TAHS in 1:1 ratio and incubated under similar conditions as the mushroom sections.

### Matrix application

Three different matrices, DHB, norharmane and NEDC were employed for the detection of lipids in both positive and negative ion mode and metabolites in negative ion mode respectively. DHB was used to spray-coat TAHS-derivatized tissue sections. The sections were dried in a vacuum desiccator prior to matrix application. The different matrices were all applied using the HTX M3 + sprayer (HTX imaging LLC, Carrboro, NC, USA).

2,5-DHB was employed for positive mode lipid analysis on untreated and derivatized mushroom sections, and amino acid standards spotted on ITO slide. A DHB solution (15 mg/mL in chloroform: methanol, 2:1 v:v) was sprayed and 15 matrix layers were applied at a flow rate of 120 µL/min. The spray nozzle was heated to 50 ℃ and the velocity of the nozzle was set at 1200 mm/min. A track spacing of 3 mm and drying time of 30 s was used. Negative ion mode lipid distributions were studied using Norharmane as a matrix. A Norharmane solution (7 mg/mL in chloroform: methanol, 2:1 v:v) was sprayed on the surface of an ITO mounted mushroom section. Twelve matrix layers were applied at a flow rate of 120 µL/min. The spray nozzle was heated to 30 ℃ and the velocity of the nozzle was set at 1200 mm/min. A track spacing of 3 mm and drying time of 30 s was used unless otherwise noted. The matrix used for the study of metabolites in negative mode was NEDC. A NEDC solution (7 mg/mL in methanol:water, 7:3 v:v) was sprayed as described elsewhere (Andersen et al. 2020) Eleven matrix layers were applied at a flow rate of 120 µL/min. The spray nozzle was heated to 75 ℃ and the velocity of the nozzle was set at 1200 mm/min.

### Mass spectrometry imaging acquisition

A Bruker rapifleX MALDI Tissuetyper time-of-flight mass spectrometer (Bruker Daltonik, Bremen, Germany) was used. Images were generated with a 50 × 50 μm pixel size. The measurements were performed using reflectron mode with 200 shots per pixel at a laser frequency of 10 kHz. MSI data was acquired with a mass range of *m/z* 100–1000 for lipids in both positive and negative ion mode and *m/z* 40–1000 for metabolites in negative ion mode.

As the rapifleX offers high speed data acquisition but lacks sufficiently high mass resolution required to differentiate between specific amino acid masses, the measurements were complemented with matrix assisted laser desorption/ionization-Fourier transform-ion cyclotron resonance mass spectrometry imaging (MALDI-FTICR-MSI) (solariX 9.4 T, Bruker Daltonics, Bremen, Germany). High mass resolution data was acquired in a mass range of *m/z* 100–600 in positive ion mode with a 75 µm spatial raster width, 250 shots per pixel, and a laser frequency of 2 kHz. Pre-defined matrix peaks were selected as reference for online lock mass calibration.

### Data Analysis and identification

The rapifleX data was imported into SCiLS Lab (MVS Version 2022a, Bruker Daltonik, Bremen, Germany) and normalized to the total ion count. Subsequently the distribution of lipids and metabolites was investigated. For amino acid investigation at high mass resolution, the solariX data was imported in SCiLS Lab and the *m/z* values of interest were matched against the list of amino acids *m/z* values reported previously by (Arts et al. 2017).

Targeted MALDI-MS/MS experiments were performed using a high mass resolution Q Exactive HF Hybrid Quadrupole-Orbitrap (Thermo Fisher Scientific GmbH, Bremen, Germany) coupled to a MALDI-ESI injector (Spectroglyph, LLC, Kennewick, WA, USA). The samples were prepared as mentioned in the MSI acquisition section, the *m/z* values of interest were manually targeted for tandem MS and 20 spectra were acquired for each precursor mass in both polarities. The injection time was set to 2,000 ms per scan. A high-energy collision dissociation cell was used, with isolation window ± 1 Da, normalized collision energy (range 10–40 manufacturer units) a laser repetition rate of 1000 Hz and mass resolution of 240,000. For amino acids, the isolation window was ± 1.3 Da, and mass resolution was 120,000. The MS/MS fragments were matched against the Human metabolome database (HMDB) (https://hmdb.ca/) and ALEX^123^ (http://www.alex123.info/) calculator for lipids and metabolites. The MS/MS fragments of derivatized mushroom sections were compared with derivatized amino acid standards.

An untargeted MALDI-MS/MS was performed to obtain maximum lipid identities possible, using an LTQ Orbitrap Elite mass spectrometer (Thermo Fischer Scientific, Bremen, Germany) in DDA (Data dependent acquisition) mode as described by Ellis et al. (Ellis et al. 2018). The samples were prepared as mentioned in the MSI acquisition section. The acquisition was performed in both negative and positive ion mode with a mass range of *m/z* 100–1000, pixel size 25 × 50 µm, mass resolution 240,000 and an injection time of 250 ms. The measurements were performed on a region of interest (ROI) selected from one of the two symmetrical halves obtained by dividing the mushroom section along the vertical plane. The data obtained was converted to imzml format, imported in Lipostar MSI software (version 1.3.1b7 Molecular Horizon, Montelino, Italy), and matched against LIPID MAPS database with automatic approval set to 3 and 4 stars.

## Results and discussion

### Optimization of sample preparation method

The Stamps/molecular imprints, fresh frozen mushroom sections, and heat-treated sections were obtained (see figure S1).

### Mushroom stamping

**Fig. 1:**
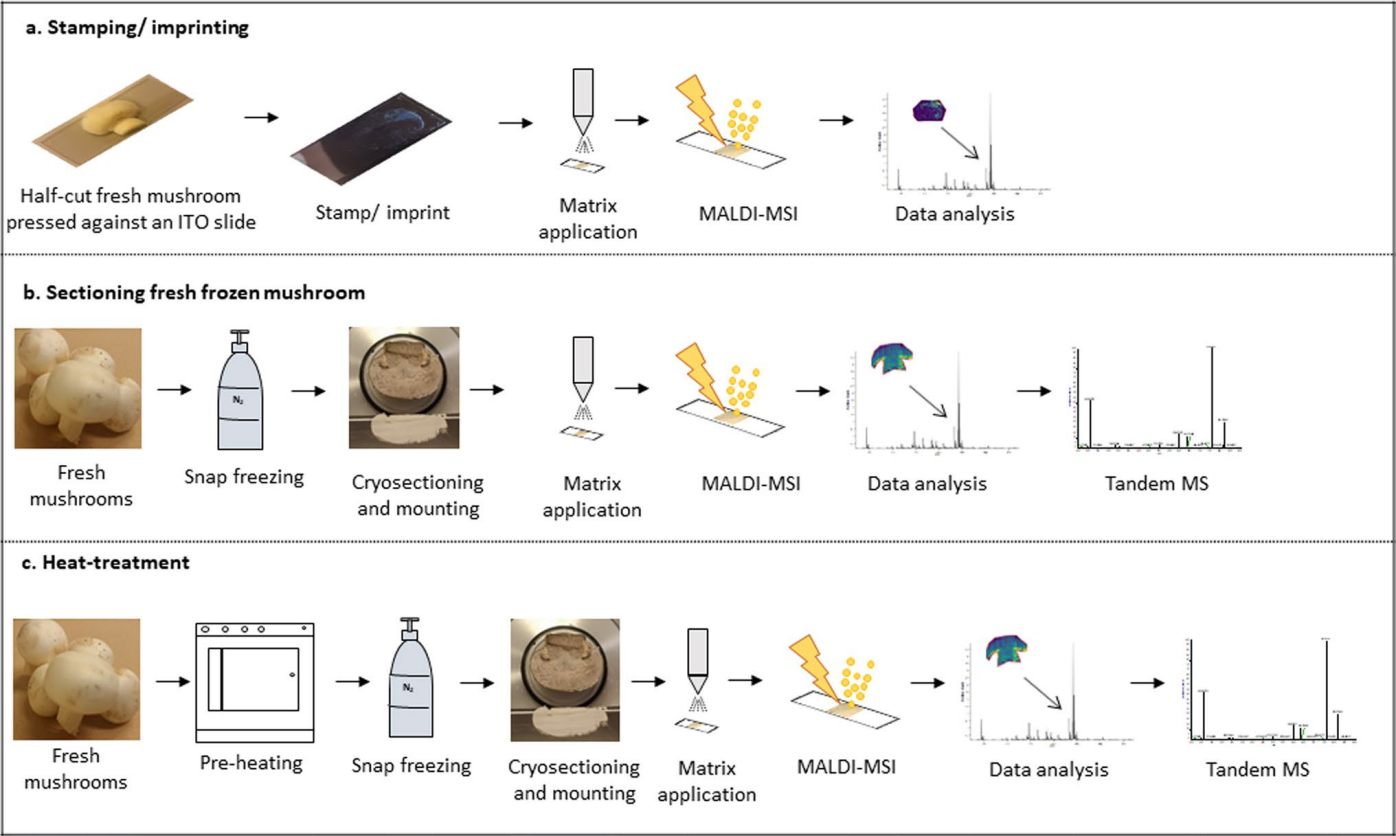
Sample preparation workflows **a** workflow of stamping/molecular imprint preparation of fresh frozen mushrooms, **b** Sectioning fresh frozen mushrooms **c** sample preparation after heat-treatment

MSI measurements were performed on the matrix covered molecular imprints/stamps of half mushrooms. (See Fig. 1A). The manual stamping approach resulted in uneven imprints as part of the mushroom surface experienced different contact pressure while pressing and lifting. It was also observed that, after stamping a small part of tissue gets attached to the slide, which causes substantial height differences. The irreproducible MS based molecular images rendered the stamping method unsuitable for routine analysis of mushrooms using MALDI-MSI and was abandoned.

### Mushroom sectioning

Targeted the optimization of the fresh frozen mushrooms through cryosectioning. In the optimized method, 35 µm thick sections were obtained (see Fig. 1B) on ITO support slides. The high water content in mushrooms resulted in ice crystals during snap freezing, therefore, at lower section thickness, it was not possible to obtain a complete intact section consisting of cap, gills and stipe. Previously, a small part of a mushroom was sliced at approx 1 mm, to analyze infections caused by soft rot pathogen on *Agaricus bisporous* (Graupner et al. 2012). Similar high water content samples, such as strawberry and bilberry, were also analyzed using MALDI-MSI. However, in these studies, the strawberry sections were 80 µm and bilberry sections were 50 µm thick and embedded in CMC (Dare et al. 2022; Enomoto et al. 2018). In the present study, sectioning was performed at 35 µm that maintained the integrity of sample without embedding.

### Heat-treatment

As mushrooms are mostly consumed after heating, which is known to dramatically change the flavor profile, mushrooms were heat-treated at 140℃ to mimic cooking conditions (see Fig. 1 (C)). The heat-treatment would help in understanding the change in distribution of flavor compounds. Moreover, the release of water not only caused migration of compounds but also change the consistency of the mushroom, making its consistency similar to a biomedical tissue. This helped to achieve a section thickness of 17 µm, which implies that, for certain food analyses, sections at lower thickness could be obtained after heat treatment as part of the sample preparation protocol.

**Fig. 2:**
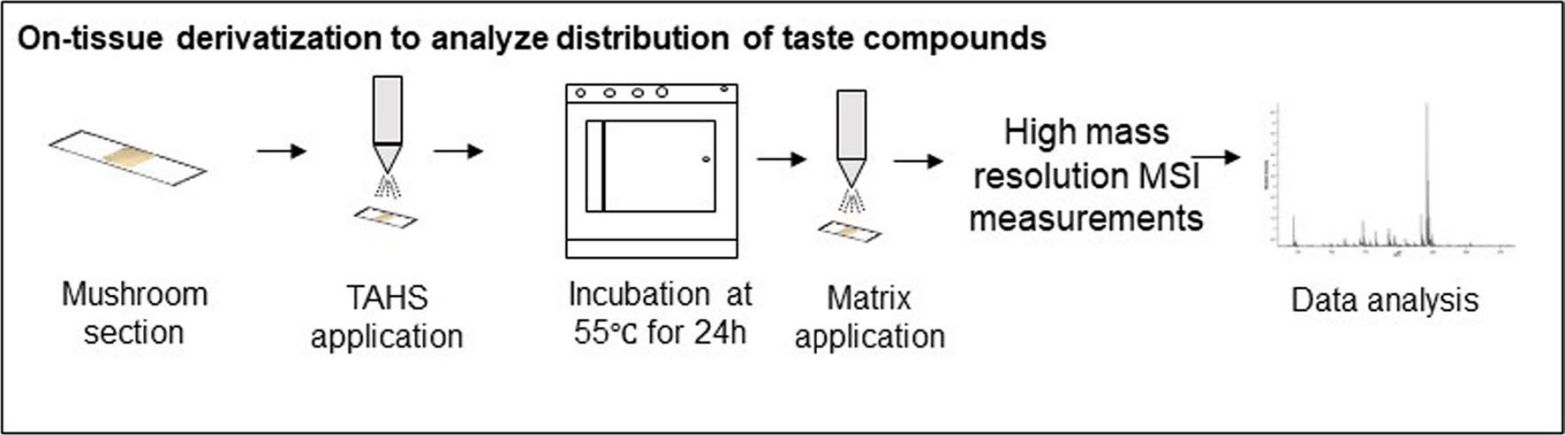
On-tissue derivatization protocol for detecting taste related amino acids in fresh and heat-treated mushroom sections

On-tissue derivatization (see Fig. 2) using TAHS is one of the chemical derivatization approaches performed to specifically enhance ionization of amino acids that are difficult to detect with other spatial biology approaches. Moreover, incubation of the derivatization agent for 24 h under humid conditions was optimal for balancing detection sensitivity and interference of high intensity matrix peaks. The derivatization process introduced a fixed positive charge at each amine functionality across the mushroom section. After the application of the DHB matrix, the exact masses of derivatized amino acids were measured using a high mass resolution solariX instrument.

### Distribution and identification of lipids and metabolites

**Fig. 3:**
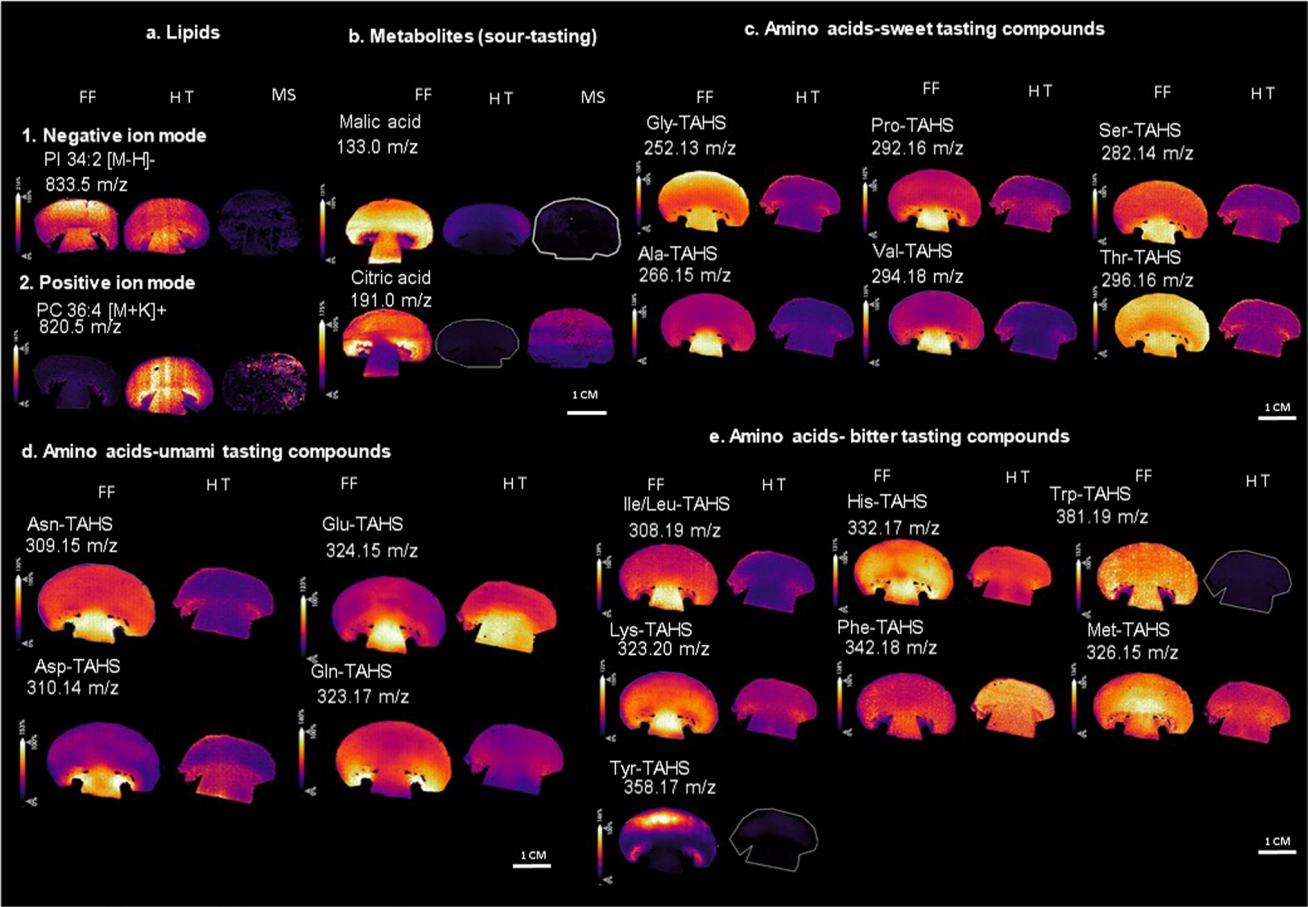
MALDI-MSI distribution of lipids and metabolites in fresh, heat-treated and stamp of mushrooms **a**-**b**. (a(1)) shows the distribution of PI 34:2[M-H]- in fresh frozen, heat-treated and stamp of mushroom (a(2)) shows the distribution of PC 36:4 [M + K] + , **b** shows malic acid distribution. **c** shows amino acids associated to sweet tasting compounds, **d** amino acids associated to umami tasting compounds- and **e** amino acids associated to bitter tasting compounds. FF refers to fresh frozen, HT refers to heat-treated mushrooms and MS refers to Mushroom stamp

Lipid and metabolite distributions were detected, imaged, and identified after optimization of the sample preparation protocol. The distributions of lipids and metabolites are shown in Fig. 3A and figure S6 A. Several lipids were identified with DDA (see table S1 and S2) in positive and negative ion mode directly on the mushroom sections, and the most abundant lipids were identified to be PC 34:2 (*m/z* 796.5) and PC 36:4 (*m/z* 820.5) and PI 34:2 (*m/z* 833.5). These lipids show a specific distribution in distinct anatomical areas of fresh frozen and heat-treated mushroom.

Metabolites detected with the NEDC matrix in negative ion mode are responsible for different flavors such as, malic acid (*m/z* 133.0) and citric acid (*m/z* 191.0) associated with a sour taste and aspartic acid (*m/z* 132.0), glutamic acid (*m/z* 146.0) and glutamine (*m/z* 145.0) are umami taste compounds. The distribution of these metabolites in fresh frozen and heat-treated mushrooms is shown in Fig. 3b and supplementary figure S6 (b) and table S3. In addition, the distribution of metabolites is studied in both fresh frozen and heat-treated mushroom sections. The analysis was extended to include golden chanterelle sections sprayed with the NEDC matrix, leading to the detection and imaging of the metabolites discussed earlier. The comparison of mass spectrum obtained with white mushrooms and chanterelle demonstrated of similar and differential compounds (figure S7 and S8). For studying the distribution of several amino acids that are responsible for flavor, on-tissue derivatization was performed using TAHS.

### On-tissue derivatization for identification of amino acids corresponding to flavor

On-tissue derivatization with TAHS was tested to visualize the distribution of the flavor related amino acids. It is shown by (Cao et al. 2021) and (Arts et al. 2017) that on-tissue derivatization enables the detection of amino acids from biological tissue sections. TAHS derivatization enhances the ionization efficiency of amino acids by coupling a fixed charge on the molecules of interest. This method was tested on mushroom sections in the present study for the detection of amino acids involved in taste modulation. High mass resolution FT-ICR-MALDI-MSI was used for accurate mass analysis and elemental composition determination. As the amino acids are derivatized with TAHS, the *m/z* values of amino acid peak found in the spectrum has the addition of *m/z* 177.1022 Da (C_10_H_13_N_2_O). For example Glycine with an addition of TAHS resulted in a peak observed at *m/z* 252.1344 [M + TAHS]^+^. Tandem MS experiments with amino acid standards and mushroom sections helped to confirm the identity of the amino acids. Moreover, the TAHS adduct was found at *m/z* 177.1022 as a fragment in all the samples during MS/MS measurements (see supplementary table S4).

The flavor compounds such as glutamine, glutamic acid, valine, aspartic acid and histidine were identified. Their distributions were visualized in both fresh frozen and heat-treated mushroom sections (see Fig. 3c-e). For example, umami and sweet tasting compounds were predominantly located in the stipe and gills in fresh frozen mushroom, which means the stipes and gills could be used for extracting the compounds responsible for these flavors. Additionally, we were able to show that amino acids were migrating after heating mostly towards gills, and cap. This information can be used by food industries to extract these flavor compounds from specific structures of fresh *Agaricus bisporous.* Extraction of flavor compounds from mushrooms has several advantages, as it is environment-friendly, uncontaminated and provides a natural way of improving the flavor profile of food items and ingredients. On-tissue derivatization using TAHS proved to be an appropriate method, to study the localization of taste compounds in mushrooms. Moreover, some of the umami flavor compounds such as aspartic acid, glutamine and glutamic acid, all detected with the NEDC matrix, showed similar distributions after on-tissue derivatization. This demonstrates that on-tissue derivatization is an appropriate method for the local detection of amino acids on fungal surfaces as it preserves their spatial distribution during the derivatization process.

## Conclusion

In this study, we have developed and optimized a sample preparation method to obtain integral thin sections of *Agaricus bisporous.* Several methods were compared and evaluated. Thin mushroom cryosectioning was determined to be the optimal procedure among those evaluated. The optimized method maintains the integrity and spatial distribution of flavor compounds in their natural anatomical location. Furthermore, pre-treatment of mushrooms at 140℃ in an oven, followed by snap freezing reduced the brittleness of mushrooms, allowed sectioning at 17 µm and aided in visualizing the localization of flavor compounds after heating. MALDI-MSI was used to study the distribution of lipids, metabolites and derivatized amino acids in mushroom sections corresponding to umami, bitter, sweet and sour tasting compounds such as lysine, glycine, asparagine, glutamine, glutamic acid, tryptophan, phenylalanine, serine and valine. The knowledge gained about the spatial distribution of taste compounds will help the food industry to modify the flavor profile in order to improve the experience of consuming mushrooms. Moreover, the flavor compounds obtained from mushrooms are natural flavor enhancers and free of contaminants. The sample preparation method described for button mushrooms is equally applicable to other types of mushrooms and could be extended to the study of other food sample types with a significant water content with MSI. Furthermore, the food industry potentially benefits from the information encoded in the spatial distribution of flavor compounds as this approach allows the adaptation of the local flavor profile of mushrooms and other fungi and use the extracted flavor compounds to improve the flavor profile of other foods.

### Supplementary Information

Below is the link to the electronic supplementary material.

Supplementary file1 (PDF 2020 kb)

## Data Availability

Available on request.

## Abbreviations

CMC: Carboxymethyl cellulose
DDA: Data dependent acquisition
DHB: 2, 5-Dihydroxybenzoic acid
GC/MS: Gas chromatography/mass spectrometry
HMDB: Human metabolome database
ITO: Indium tin oxide
LAESI: Laser ablation electrospray ionization
LA-ICP-MS: Laser ablation inductively coupled mass spectrometry
LC–MS: Liquid chromatography- mass spectrometry
m/z: Mass-to-charge-ratio
MALDI-ESI: Matrix assisted laser desorption/ ionization –electrospray ionization
MALDI-FTICR-MSI: Matrix assisted laser desorption/ ionization Fourier transform ion cyclotron resonance mass spectrometry imaging
MALDI-MSI: Matrix assisted laser desorption/ionization mass spectrometry imaging
MALDI-TOF: Matrix assisted laser desorption/ ionization-Time of flight
MSI: Mass spectrometry imaging
NEDC: N-(1-naphthyl) ethylenediamine dihydrochloride
NMR: Nuclear magnetic resonance
TAHS: P-N,N,N- trimethylamonioanilyl N-hydroxysuccinimidylcarbamate iodide
