# Supplementary material for: Mass spectrometry imaging reveals flavor distribution in edible mushrooms
Source: J Food Sci Technol. 2023 Nov 13;61(5):888–96. doi: 10.1007/s13197-023-05883-0 (PMC10933231; doi:10.1007/s13197-023-05883-0)
Supplement: Supplementary file 1 — Supplementary file1 (PDF 2020 kb) [file 13197_2023_5883_MOESM1_ESM.pdf]

Supplementary information

Mass spectrometry imaging reveals flavor distribution in edible mushrooms

Contents

Figure S1: Optical images of sample preparation methods: stamping and sectioning

Figure S2: Mass spectra of lipids and metabolites in fresh frozen mushrooms.

Figure S3: Mass spectra of lipids and metabolites in pre-heated mushrooms.

Figure S4: Mass spectra of lipids and metabolites in mushroom stamp.

Figure S5: Mass spectra of amino acids in fresh frozen Pre-heated mushrooms.

Figure S6: MALDI-MSI images of lipids and metabolites.

Figure S7. MALDI-MSI mass spectrum of metabolites in chanterelle mushrooms v/s white button mushrooms after heat-treatment

Figure S8. MALDI-MSI images of metabolites in chanterelle mushroom sections after heat-treatment

Table S1: Tandem MS of lipids in positive ion mode

Table S2: Tandem MS of lipids in negative ion mode

Table S3: Tandem MS of metabolites

Table S4: Identification of on-tissue derivatized amino acid and the standards

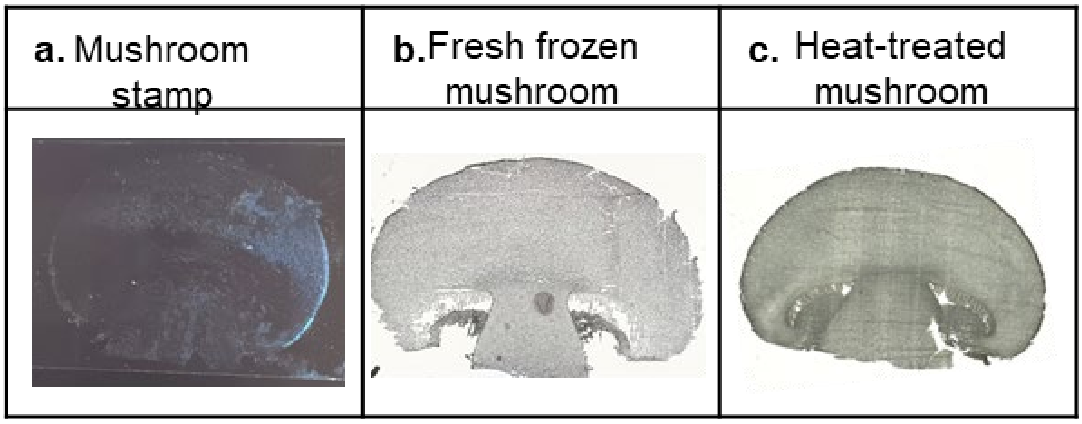

Figure S1: Optical images of (A) Mushroom stamp, (B) fresh frozen section of 35 µm and (C) Heat-treated mushroom section of 17 µm

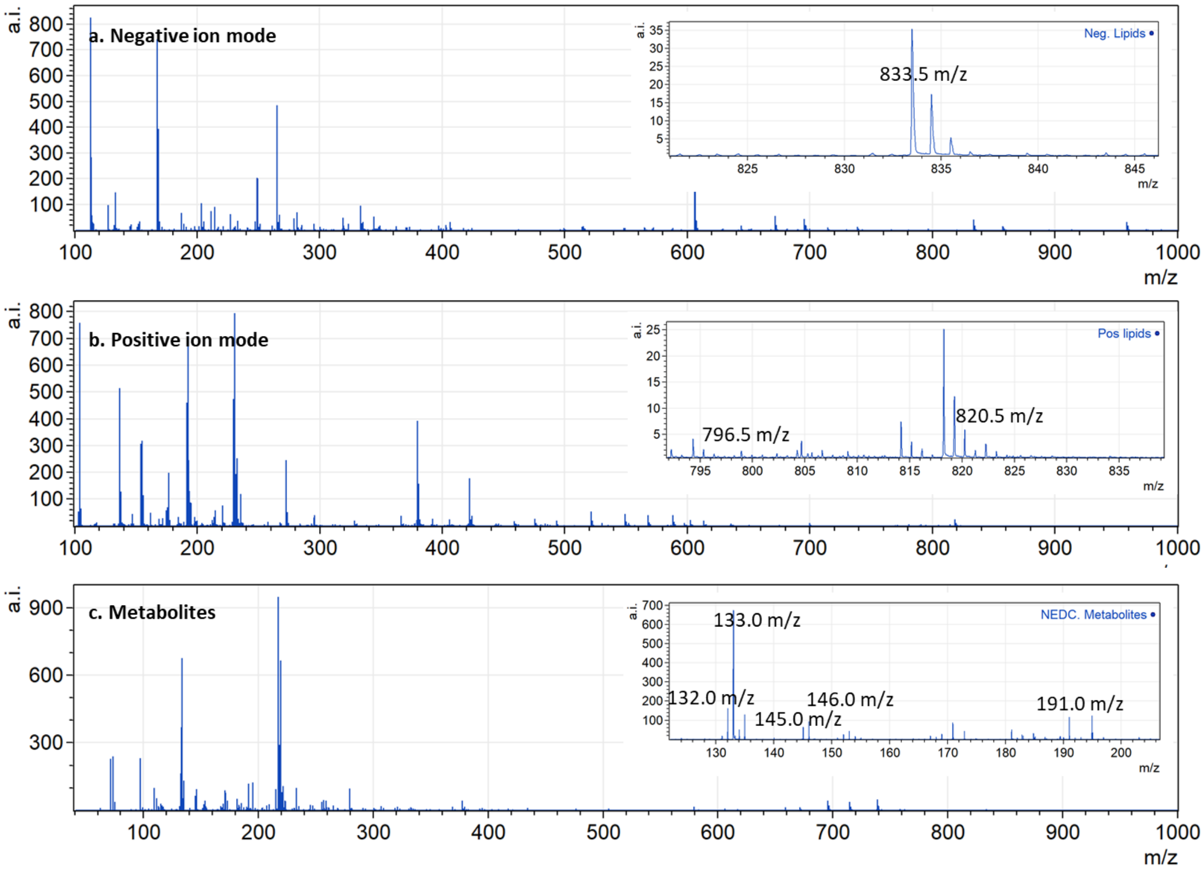

Figure S2. Mass spectra of lipids and metabolites in Fresh frozen mushrooms. Full range (100-1000 m/z) mass spectra with an insertion of zoom in having identified compounds (A) lipids in negative ion mode with norharmane, (B) lipids in positive ion mode with DHB, and (C) metabolites in negative ion mode with NEDC.

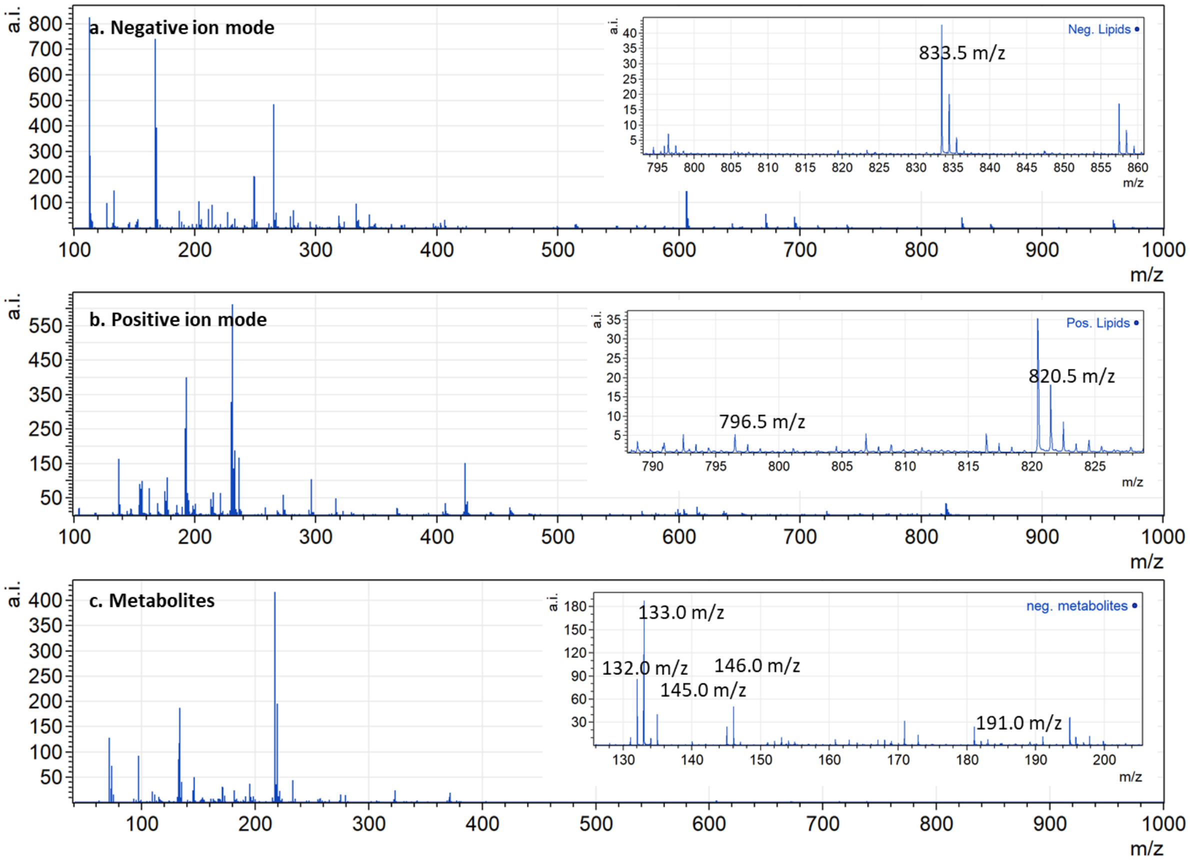

Figure S3. Mass spectra of lipids and metabolites in pre-heated mushrooms. Full range (100-1000 m/z) mass spectra with an insertion of zoom in having identified compounds (A) lipids in negative ion mode with norharmane, (B) lipids in positive ion mode with DHB, and (C) metabolites in negative ion mode with NEDC

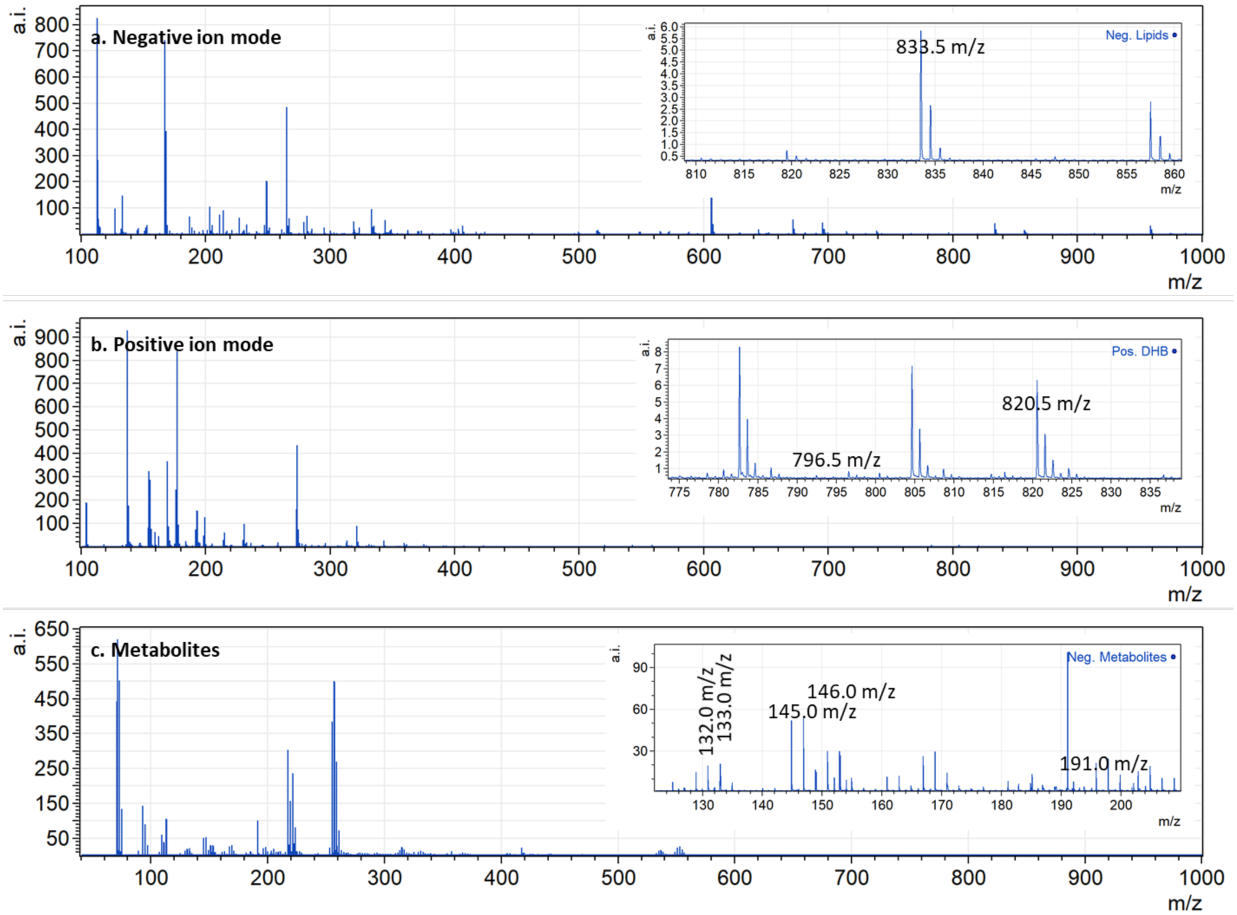

Figure S4. Mass spectra of lipids and metabolites in mushroom stamp. Full range of lipids (100-1000 m/z) and metabolites (40-1000 m/z) mass spectra with an insertion of zoom in having identified compounds (A) lipids in negative ion mode with norharmane, (B) lipids in positive ion mode with DHB, and (C) metabolites in negative ion mode with NEDC

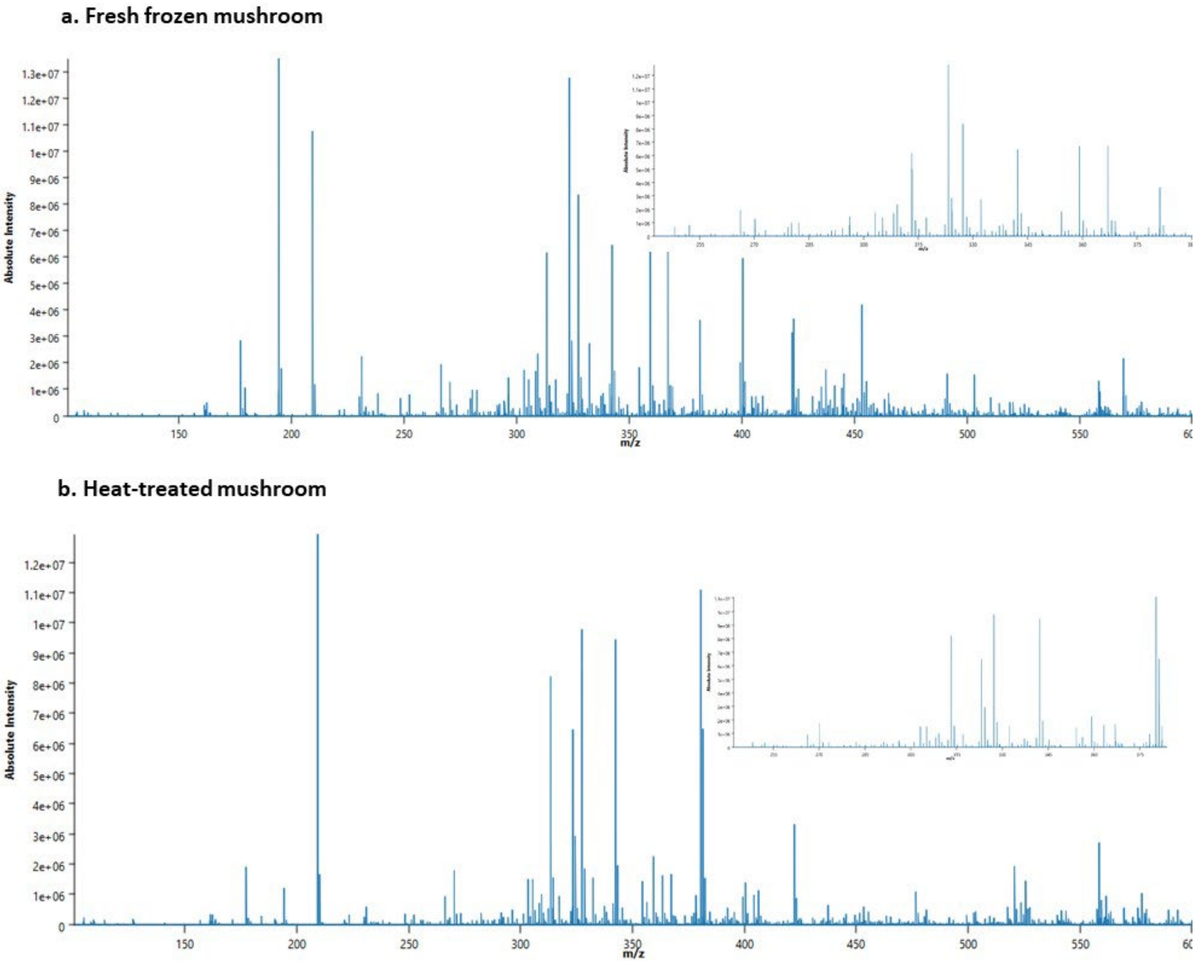

Figure S5: Mass spectra of amino acids in a)fresh frozen and b) pre-heated mushrooms. Inset shows zoomed spectra in the range from 240 to 390 m/z

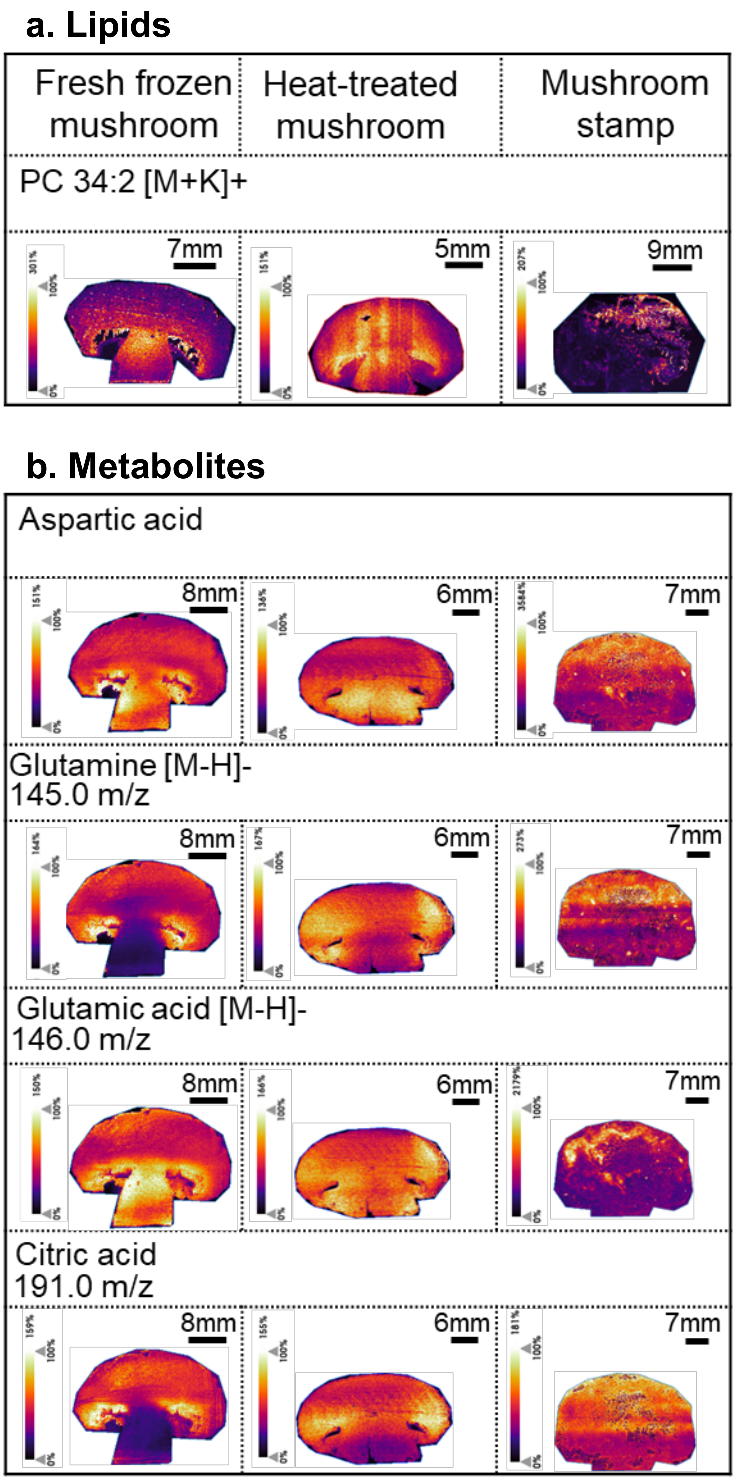

Figure S6. Overview of MALDI-MSI selected ion images of a) the lipid PC32:4 and b) selected metabolites

Name of compound	m/z of Compound	Chemical Formula	Lipid Class	Adducts	Mass error (ppm)	Fragment 1	Fragment 2
PC 18:2	520.3399	C26H50NO7P	Monoacylglycerophosphocholines	[M+H]+	0.2	104.1069	184.0733
PE 36:4	778.4783	C41H74NO8P	Diacylglycerophosphoethanolamines	[M+K]+	0.1	637.4592	599.5033
PC 34:2	796.5252	C42H80NO8P	Diacylglycerophosphocholines	[M+K]+	0.2	613.4592	737.4518
PC 36:4	804.5512	C44H80NO8P	Diacylglycerophosphocholines	[M+Na]+	0.3	621.4853	745.4779
PC 36:4	820.5253	C44H80NO8P	Diacylglycerophosphocholines	[M+K]+	0	637.4592	761.4518
PC 38:2	852.5877	C46H88NO8P	Diacylglycerophosphocholines	[M+K]+	0.3	669.5219	793.5144
PC 40:2	880.6189	C48H92NO8P	Diacylglycerophosphocholines	[M+K]+	0.2	697.5532	821.5457

Table S1: Tandem MS of lipids in positive ion mode

Name of compound	m/z of Compound	Chemical Formula	Lipid Class	Adducts	Mass error (ppm)	Fragment 1	Fragment 2
PI 18:2	595.289	C27H49O12P	Monoacylglycerophosphoinositols	[M-H]-	0.3	279.2329	241.0118
PA 18:2	433.2356	C21H39O7P	Monoacylglycerophosphates	[M-H]-	1.1	279.2329	152.9958
PA 34:2	671.465	C37H6908P	Diacylglycerophosphates	[M-H]-	1.1	279.2329	255.2329
PA 36:4	695.4649	C39H69O8P	Diacylglycerophosphates	[M-H]-	1.1	303.2329	255.2329
PA 38:5	721.4806	C41H71O8P	Diacylglycerophosphates	[M-H]-	1.1	331.2642	253.2173
PE 36:2	742.5384	C41H78NO9P	Diacylglycerophosphoethanolamines	[M-H]-	1.1	281.2486	478.2939
PE 34:2	714.5071	C39H74NO8P	Diacylglycerophosphoethanolamines	[M-H]-	1.2	279.2329	452.2782
PE 36:4	738.5071	C41H74NO8P	Diacylglycerophosphoethanolamines	[M-H]-	1.2	281.2486	277.2173
PE 38:4	766.5382	C43H78NO8P	Diacylglycerophosphoethanolamines	[M-H]-	1.3	309.2799	277.2173
PI 32:2	805.4862	C41H75O13P	Diacylglycerophosphoinositols	[M-H]-	1.3	241.0118	281.2486
PS 34:2	758.4967	C40H74NO10P	Diacylglycerophosphoserines	[M-H]-	1.4	671.4657	295.2642
PI 34:2	833.5174	C43H79O13P	Diacylglycerophosphoinositols	[M-H]-	1.4	279.2329	241.0118
PI 36:4	857.5173	C45H79O13P	Diacylglycerophosphoinositols	[M-H]-	1.5	279.161	415.159
PI 36:2	861.5486	C45H83O13P	Diacylglycerophosphoinositols	[M-H]-	1.5	581.198	419.175

Table S2: Tandem MS of lipids in negative ion mode

Name	Chemical formula	Theoretical m/z	Observed m/z	Mass error (ppm)	Fragment 1	Fragment 2
Glutamic acid	$C_5H_9NO_4$	146.0458	146.0459	0.13	128.0354	102.0561
Glutamine	$C_5H_{10}N_2O_3$	145.0607	145.0618	-0.176	127.0513	129.4133
Aspartic acid	$C_4H_7NO_4$	132.0291	132.0302	-0.159	113.7352	88.0404
Malic acid	$C_4H_6O_5$	133.0131	133.0143	0.026	115.0036	71.0137
Citric acid	$C_6H_8O_7$	191.0186	191.0198	0.755	129.0193	87.0088

Table S3: Tandem MS of metabolites in negative ion mode

Compound	Chemical formula [aa + TAHS]	m/z Theo	Standards		Fresh mushroom		Pre-heated mushroom	
			m/z obs	Mass error (ppm)	m/z obs	Mass error (ppm)	m/z obs	Mass error (ppm)
Gly	C12 H18 O3 N3	252.1343	252.1344	0.444	252.1343	0.32	252.13411	-0.62
Ala	C13 H20 O3 N3	266.1499	266.1499	-0.406	266.1499	-0.03	266.1497	-0.81
Ser	C13 H20 O4 N3	282.1448	282.1443	-1.781	282.1448	-0.11	282.1446	-0.96
Val	C15 H24 O3 N3	294.1812	294.1811	-0.402	294.1812	0.04	294.1806	-1.79
Thr	C14 H22 O4 N3	296.1604	296.1604	-0.178	296.1605	0.12	296.1602	-0.75
Ile/leu	C16 H26 O3 N3	308.1968	308.1967	-0.611	308.1968	0	308.1964	-1.61
Asn	C14 H21 O4 N4	309.1557	309.1557	-0.232	309.1555	-0.52	309.155	-2.27
Lys	C16 H27 O3 N4	323.2078	323.2076	-0.455	323.2078	0.009	323.2075	-0.92
Glu	C15 H22 O5 N3	324.1554	324.1552	-0.54	324.1554	-0.11	324.1551	-0.82
Met	C15 H24 O3 N3 S	326.1533	326.1532	-0.210	326.1533	0.24	326.1531	-0.67
His	C16 H22 O3 N5	332.1717	332.1713	-1.102	332.1716	-0.22	332.1714	-1.04
Pro	C15 H22 O3 N3	292.1656	-	-	292.1655	-0.23	292.1652	-1.26
Asp	C14 H20 O5 N3	310.1397	-	-	310.1396	-0.24	310.1394	-1.02
Gln	C15 H23 O4 N4	323.1714	-	-	323.1714	-0.06	323.1711	-0.99
Phe	C19 H24 O3 N3	342.1812	-	-	342.1812	0.09	342.181	-0.69
Tyr	C19 H24 O4 N3	358.1761	-	-	358.176	-0.45	-	-
Trp	C21 H25 O3 N4	381.1921	-	-	381.192	-1.03	381.1917	-1.04

Table S4: Identification of on-tissue derivatized amino acid and the standards

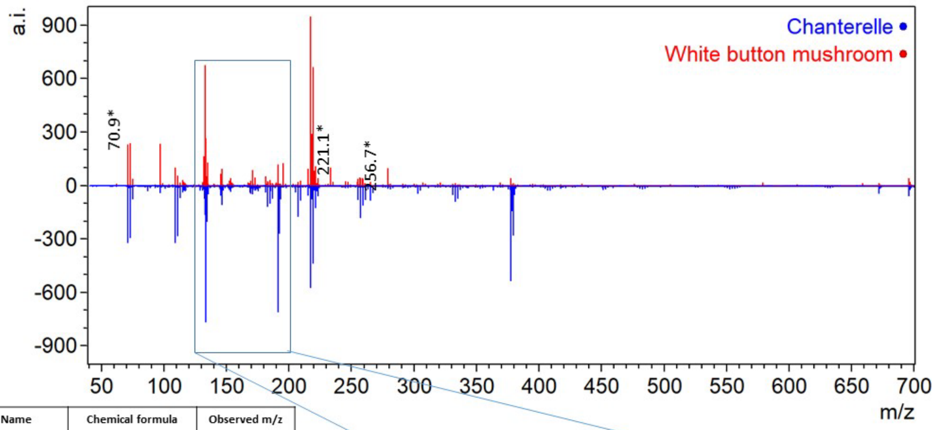

Name	Chemical formula	Observed m/z
Glutamic acid	$C_5H_9NO_4$	146.0
Glutamine	$C_5H_{10}N_2O_3$	145.0
Aspartic acid	$C_4H_7NO_4$	132.0
Malic acid	$C_4H_6O_5$	133.0
Citric acid	$C_6H_8O_7$	191.0

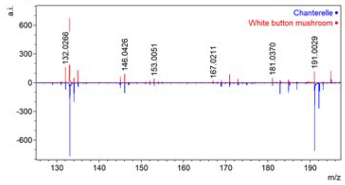

MALDI-ToF-MSI- Rapiflex
Ion mode: Negative
Matrix: NEDC (70% MeOH)
Spatial resolution: 50 µm
Mass range: m/z 40-1000

* Matrix peaks

Figure S7. MALDI-MSI mass spectrum of metabolites in chanterelle mushrooms v/s white button mushrooms after heat-treatment

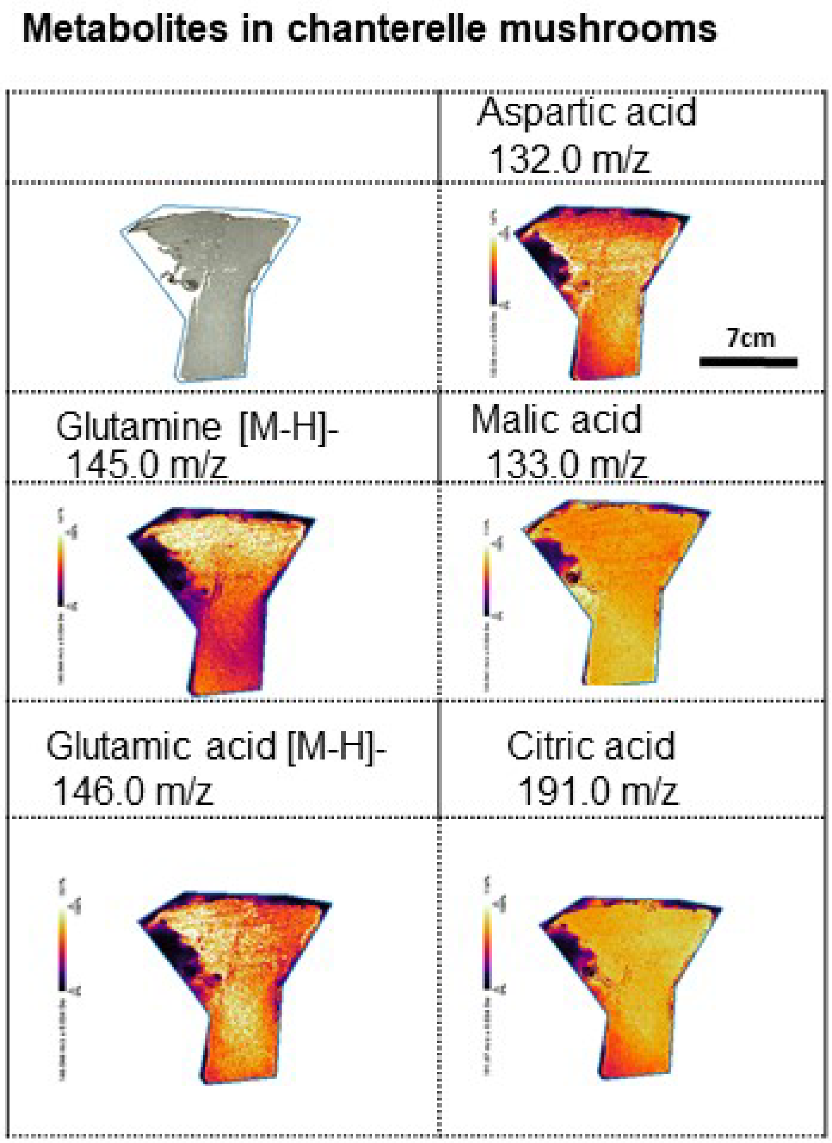

Figure S8. MALDI-MSI images of metabolites in chanterelle mushroom sections after heat-treatment
